# Worsening of health disparities across COVID-19 pandemic stages in Korea

**DOI:** 10.4178/epih.e2024038

**Published:** 2024-03-13

**Authors:** Hyejin Lee, Hyunwoo Nam, Jae-ryun Lee, Hyemin Jung, Jin Yong Lee

**Affiliations:** 1 Department of Family Medicine, Seoul National University Bundang Hospital, Seongnam, Korea; 2 Department of Family Medicine, Seoul National University College of Medicine, Seoul, Korea; 3 Seoul National University College of Medicine, Seoul, Korea; 4 Department of Education and Human Resource Development, Seoul National University Hospital, Seoul, Korea; 5 Public Health Care Center, Seoul National University Hospital, Seoul, Korea; 6 Department of Health Policy and Management, Seoul National University College of Medicine, Seoul, Korea; 7 Institute of Health Policy and Management, Seoul National University Medical Research Center, Seoul, Korea

**Keywords:** COVID-19, Disparity, National Health Insurance, Mortality, Case-fatality

## Abstract

**OBJECTIVES:**

With the end of the coronavirus disease 2019 (COVID-19) pandemic, the health outcomes of this disease in Korea must be examined. We aimed to investigate health outcomes and disparities linked to socioeconomic status during the COVID-19 pandemic in Korea and to identify risk factors for hospitalization and mortality.

**METHODS:**

This nationwide retrospective study incorporated an analysis of individuals with and without COVID-19 in Korea between January 1, 2020 and December 31, 2022. The study period was divided into 4 stages. Prevalence, hospitalization, mortality, and case-fatality rates were calculated per 100,000 population. Multivariate logistic regression was performed to identify risk factors for COVID-19 hospitalization and mortality.

**RESULTS:**

Overall, the incidence rate was 40,601 per 100,000 population, the mortality rate was 105 per 100,000 population, and the case-fatality rate was 259 per 100,000 cases. A total of 12,577,367 new cases (24.5%) were recorded in stage 3 and 8,979,635 cases (17.5%) in stage 4. Medical Aid recipients displayed the lowest 3-year cumulative incidence rate (32,737 per 100,000) but the highest hospitalization (5,663 cases per 100,000), mortality (498 per 100,000), and case-fatality (1,521 per 100,000) rates. Male sex, older age, lower economic status, non-metropolitan area of residence, high Charlson comorbidity index, and disability were associated with higher risk of hospitalization and death. Vaccination was found to reduce mortality risk.

**CONCLUSIONS:**

As the pandemic progressed, surges were observed in incidence, hospitalization, and mortality, exacerbating disparities associated with economic status and disability. Nevertheless, Korea has maintained a low case-fatality rate across all economic groups.

## GRAPHICAL ABSTRACT

[Fig f1-epih-46-e2024038]

## Key Message

Using data from the National Health Insurance Service, a retrospective cohort for the years 2020-2022 was established. By examining the COVID-19 prevalence rate, hospitalisation rate, mortality rate, and case-fatality rate, along with health disparities based on disability and economic status, as the pandemic progressed, there was a surge in incidence, hospitalisation, and mortality, widening disparities related to economic status and disability. Despite these disparities, Korea has maintained a low case-fatality rate across all economic groups.

## INTRODUCTION

The outbreak of coronavirus disease 2019 (COVID-19) represents one of the most notable pandemics in history [1]. The World Health Organization (WHO) declared the COVID-19 outbreak a Public Health Emergency of International Concern (PHEIC) on January 30, 2020. A pandemic was officially declared on March 11, 2020, followed by continued efforts to combat COVID-19. On May 5, 2023, the WHO lifted the PHEIC, marking the final phase of the pandemic response [2].

Responses to COVID-19 have varied across countries and over time. Some nations have implemented lockdowns, border closures, and temporary shutdowns of schools and offices, with provisions made for remote work [3]. In the early stages of the pandemic, Korea, along with the rest of the Western Pacific region—which had the lowest death rate among the WHO regions—adopted active control strategies. These included social distancing, mandatory face mask usage, and widespread testing for COVID-19 [4]. Korea managed to contain the spread of the COVID-19 virus through early adoption of the “3T” strategy (testing, tracing, and treatment), which involved measures such as testing, hospitalization, and the use of medication [5]. Moreover, Korea introduced universal health coverage (UHC) for COVID-19 at the beginning of the pandemic in 2020 and maintained it during the early stages [6]. As the pandemic progressed and vaccination programs were initiated, Korea shifted its focus to a gradual recovery strategy [7]. During this phase, the emergence of the Omicron variant (B.1.1.529) led to a rapid increase in COVID-19 cases [8].

However, the impact of changes in quarantine policies on the incidence, severity, and mortality associated with COVID-19 remains unclear. Furthermore, it is uncertain whether the COVID19 pandemic has led to health disparities based on socioeconomic status. Such disparities were not evident at the onset of the pandemic; however, they may have developed over the 3-year course of the pandemic [6].

Consequently, this study was conducted to examine the health outcomes of the COVID-19 pandemic in Korea over time, as well as to ascertain the existence of health disparities based on socioeconomic status. Additionally, we sought to identify risk factors associated with hospitalization and mortality due to COVID-19.

## MATERIALS AND METHODS

### Study design, setting, and population

In this nationwide retrospective study, we examined a cohort of individuals both with and without COVID-19 in Korea, segmenting the timeframe from January 1, 2020 to December 31, 2022, into 4 distinct stages. The dataset utilized for this research was the Korea Disease Control and Prevention Agency-COVID-19- National Health Insurance Service (K-COV-N) cohort, supplied by the Korean National Health Insurance Service (NHIS). This dataset encompasses nearly the entire Korean population and includes records of domestic medical utilization. The data include detailed information on medical services used, COVID-19 vaccination status, diagnostic records, and dates of death from the national death registry. As of January 1, 2020, the study included 51,984,158 members of the general population. Across the study period, 21,105,865 of these patients were infected with COVID-19 at least once, while 30,878,293 individuals had no recorded infection with the virus.

### Stages

In this cross-sectional study, the study period was divided into 4 stages, each corresponding to a phase of the COVID-19 outbreak and ensuing national response. The timeframes for the stages were as follows: stage 1 spanned from January 1, 2020 to February 28, 2021; stage 2 from March 1, 2021 to October 31, 2021; stage 3 from November 1, 2021 to May 31, 2022; and stage 4 from June 1, 2022 to December 31, 2022.

During stage 1—the initial response period for COVID-19— the first confirmed case in Korea was reported on January 20, 2020 [9]. Subsequently, community transmission was confirmed, which led to the issuance of an infectious disease crisis alert at the “serious” level on February 23, 2020 [10]. In response, social distancing measures were implemented, including limitations on gatherings, and an executive order mandating the use of masks was issued.

During stage 2, social distancing and quarantine measures were intensified, and vaccination programs were launched. These programs began to roll out across Korea on February 26, 2021 [11]. Social distancing measures reached their peak on July 12, 2021, in response to the widespread transmission of the Delta variant beginning in May 2021. Restrictions included a ban on private gatherings of 3 or more people, limitations on commuting, and curfews. Additionally, venues such as restaurants and gyms were either closed or subjected to reduced operating hours [12].

During stage 3, attempts were made to ease social distancing measures. Patients with severe illness were hospitalized, and high-risk groups were closely monitored. The government shifted its strategy to a gradual return to normalcy, which included easing social distancing, streamlining quarantine protocols, and reducing the duration of self-isolation (November 2021). The Omicron variant was first detected domestically in December 2021 and became the dominant strain in 2022 [13].

Stage 4 marked the period of full day-to-day recovery, wherein most mandatory quarantine measures were lifted, apart from mask-wearing. The vaccination completion rate surpassed 85%, and both the obligatory quarantine period and COVID-19 testing of overseas arrivals were discontinued [11].

### Variables

Patients with COVID-19 were identified by diagnosis codes, specifically B34.2, B97.2, U18, U18.1, and U07.1. These codes correspond to the seventh edition of the Korean Standard Classification of Diseases and Mortality, adapted from the 10th edition of the International Classification of Diseases. Economic status was categorized into 4 levels based on health insurance premiums, which are indicative of the economic status of health insurance beneficiaries. A fifth category included Medical Aid recipients. Geographic areas were classified as either metropolitan or nonmetropolitan. Comorbidity was quantified as a single integer using the Charlson comorbidity index (CCI), a measure of disease burden that predicts mortality risk [14]. Disability status was determined using data from the National Disability Registry. Vaccination was considered complete if the individual had received 2 or more doses of most vaccines or 1 dose of the Janssen vaccine.

Although not included in the analysis, we confirmed the presence of each individual comorbidity. Hypertension (codes I10 and I15), diabetes (codes E10, E118, E119, E13, and E149), and hypercholesterolemia (code E78) were identified based on claims made over the prior 5 years. Myocardial infarction (codes I21 and I22), stroke (codes I60-63), and cancer (codes C00-97) were defined by hospitalization claims within the prior 5 years.

Among those with COVID-19, both the severity and the number of confirmed cases were analyzed. The severity of COVID-19 infection was categorized as follows: ambulatory state (categories 1-2), hospitalized mild disease (categories 3-4), and hospitalized severe disease (categories 5-7), according to the WHO’s 7-category ordinal scale for COVID-19 [15]. “Hospitalized mild disease” includes patients who require hospitalization but do not necessitate oxygen therapy or receive oxygen via a mask or nasal prongs. In contrast, “hospitalized severe disease” covers individuals who require non-invasive ventilation, high-flow oxygen, intubation, mechanical ventilation, or additional organ support. The number of confirmed COVID-19 cases was determined by reviewing the diagnoses of COVID-19 at each stage and summing the total number of diagnoses.

### Statistical analysis

We compared patient characteristics across the 4 stages. Population figures for each stage were based on the population on the first day of that stage. Baseline characteristics are presented as medians with ranges, means with standard deviations, or counts with percentages. We calculated the incidence (per 100,000), hospitalization (per 100,000), mortality (per 100,000), and case-fatality rates for each stage. The incidence, hospitalization, and mortality rates were determined by dividing the number of events by the total population. In contrast, the case-fatality rate was calculated by dividing the number of deaths among patients with COVID-19 by the number of confirmed cases during the specified stage. We aggregated individual cases to calculate the incidence, severity, and case-fatality rates for each stage. The overall period spans 3 years, with stage 1 lasting 14 months, stage 2 spanning 8 months, and stages 3 and 4 each lasting 7 months. These durations were incorporated into the cumulative incidence, severity, and case-fatality calculations.

Descriptive statistics were analyzed using the 2-tailed Student t-test or analysis of variance for continuous variables and the chisquare test for categorical variables. To identify risk factors for hospitalization and mortality, multivariate logistic regression was employed to calculate 95% confidence intervals (CIs) and adjusted odds ratios (aORs). The overall analysis was performed using the complete dataset across all stages, excluding the stage variable from the model. Separate stage-specific analyses were also conducted by categorizing the data by stage. All statistical tests were 2-tailed, and p-values less than 0.05 were considered to indicate statistical significance. SAS Enterprise Guide version 8.2 (SAS Institute Inc., Cary, NC, USA) was used for the statistical analysis.

### Ethics statement

The Institutional Review Board of Seoul National University Bundang Hospital granted approval for this research (IRB No. X-2207-768-901).

## RESULTS

Between January 1, 2020 and December 31, 2022, of 51,984,158 individuals, 21,105,865 (40.6%) had been confirmed to have COVID-19, having contracted the virus at least once. In contrast, 30,878,293 individuals (59.4%) either had not been infected or had not been confirmed to have COVID-19. Among the confirmed patients, female constituted a higher proportion than male, with 11,612,725 cases (55.0%). Regarding age, the highest number of cases was observed among those 20-44 years old, at 7,536,232 (35.7%). When categorized by socioeconomic status, those in the highest economic level were the most heavily represented among both patients with confirmed COVID-19 and those who were uninfected/unconfirmed (35.9 and 31.7%, respectively), while Medical Aid recipients had the lowest representation (2.3 and 3.2%, respectively). Across every level of economic status, the number of individuals who were not infected or confirmed surpassed the number of patients with confirmed COVID-19. The majority of both those with confirmed COVID-19 (n= 15,022,546, 71.2%) and uninfected/unconfirmed individuals (n= 21,377,186, 69.2%) resided in metropolitan areas. The number of individuals with a CCI score of 0 was 11,265,937 (53.4%) among patients with confirmed COVID-19 and 17,449,743 (56.5%) among those who were uninfected/unconfirmed. Individuals with disabilities represented a smaller proportion of the patients with confirmed COVID-19 than of uninfected/unconfirmed individuals, with 916,787 (4.3%) and 1,774,651 (5.8%) individuals, respectively. Of the total population, 42,853,865 (82.4%) people had completed the full vaccination program, with 17,213,952 (81.6%) patients with confirmed COVID-19 and 25,639,913 (83.0%) uninfected/unconfirmed individuals achieving full vaccination coverage. Individuals with confirmed COVID-19 exhibited a low incidence of comorbidities, with 20,011,285 (94.8%) individuals experiencing a non-hospitalized ambulatory state. Among these patients, 776,760 (3.7%) individuals were confirmed to be infected with the virus on 2 separate occasions, while a minority of 3,993 (0.02%) individuals were confirmed 3 or more times (Table 1).

Throughout the study period, 21,105,865 (40.6%) individuals had confirmed COVID-19, corresponding to an incidence of 40,601 per 100,000 population. The number of deaths was 54,638 (0.1%), corresponding to a mortality rate of 105 per 100,000 population, while the case-fatality rate was 259 per 100,000 population. Stage 1 included 96,889 (0.2%) new confirmed cases, while stage 2 had 236,870 (0.5%), stage 3 had 12,577,367 (24.5%), and stage 4 had 8,979,635 (17.5%). When translated to incidence rates per 100,000 people, these figures corresponded to 186, 459, 24,457, and 17,529, respectively. In terms of severity, most ambulatory cases occurred in stages 3 and 4, with incidences of 23,436 and 16,952 cases per 100,000 people, respectively. In stage 3, the mortality rate was also elevated, reaching 65 per 100,000 individuals, while in stage 4, it was slightly lower, at 35 per 100,000 individuals. Conversely, the case-fatality rate reached its peak of 1,800 per 100,000 cases in stage 1, followed by 593 per 100,000 cases in stage 2, then decreased further to 266 per 100,000 cases in stage 3 and 201 per 100,000 cases in stage 4 (Table 2).

Regarding socioeconomic status, the lowest incidence of confirmed COVID-19 cases was found among Medical Aid recipients (p< 0.001), of whom 486,971 individuals had the disease, representing 32.7% of this economic group. Interestingly, in the first stage, the highest incidence rate—328 per 100,000 population— was recorded among Medical Aid recipients (p< 0.001). However, during the second phase, the incidence rate of this group was comparable to that of the other economic levels (p< 0.001). In the third and fourth phases, the incidence rates were 21,126 and 14,508 per 100,000, respectively, which were lower than the rates observed in the other groups (p< 0.001). When considering disability status, the incidence of COVID-19 was higher among those without disabilities, with rates of 40,958 per 100,000 for individuals without disabilities and 34,064 per 100,000 for those with disabilities (p< 0.001). In the first stage, individuals with disabilities experienced a higher incidence rate; however, in the second, third, and fourth phases, the incidence was greater among those without disabilities (p< 0.001).

Hospitalization rates were highest among Medical Aid recipients, with 5,663 cases per 100,000 population (p< 0.001). Specifically, Medical Aid recipients in stages 3 and 4 faced hospitalization rates approximately 3 times to 4 times higher than those of other economic statuses (p< 0.001). Individuals with confirmed disabilities experienced a higher number of hospitalizations, at 5,639 per 100,000, compared to 1,913 for those without disabilities (p<0.001). During stages 3 and 4, the hospitalization rates for individuals with disabilities were roughly 4 times higher than for those without disabilities (p< 0.001). Additionally, the incidence of severe disease requiring hospitalization was highest among Medical Aid recipients (298 per 100,000) and individuals with disabilities (605 per 100,000), with these trends persisting across all stages (p< 0.001).

Regarding economic status, mortality (498 per 100,000) and case-fatality (1,521 per 100,000) were highest among Medical Aid recipients (p< 0.001). Additionally, individuals with disabilities displayed higher case-fatality (1,776 per 100,000) than those without disabilities. The case-fatality rate across the entire study period was approximately 6 times higher among Medical Aid recipients (representing those with the lowest economic status) than the other groups. However, when examining case-fatality by stage, distinct patterns emerged. In stage 1, the case-fatality rate was approximately 4 times higher among the Medical Aid group, whereas in stage 2 it ranged from 5 times to 6 times higher. In stage 3, the case-fatality rate increased to approximately 7 times to 8 times higher, and in stage 4 it remained consistent at about 6 times to 7 times higher. Relative to those without disabilities, individuals with disabilities faced the highest mortality and case-fatality rates during stages 3 and 4 (Table 3).

In the logistic regression analysis conducted to identify factors associated with hospitalization, female exhibited a low aOR, at 0.84 (95% CI, 0.84 to 0.84), relative to male. Higher risks of hospitalization were observed in adults aged 75 years or older (aOR, 5.17; 95% CI, 5.13 to 5.21), individuals with lower economic status (Medical Aid recipients: aOR, 2.55; 95% CI, 2.53 to 2.58), residents of non-metropolitan areas (aOR, 1.07; 95% CI, 1.06 to 1.07), those with a CCI of 3 or more (aOR, 1.59; 95% CI, 1.58 to 1.60), and individuals with disabilities (aOR, 1.85; 95% CI, 1.84 to 1.86). Completion of vaccination was associated with a lower risk of hospitalization, with an aOR of 0.40 (95% CI, 0.39 to 0.40) compared to those with incomplete vaccination. The aOR for individuals aged 75 years or older was 2.06 (95% CI, 1.92 to 2.21) in stage 1, 0.99 (95% CI, 0.91 to 1.07) in stage 2, 6.91 (95% CI, 6.84 to 6.98) in stage 3, and 9.32 (95% CI, 9.19 to 9.45) in stage 4, indicating that the risk of hospitalization for older adults increased in stages 3 and 4. The higher aOR for hospitalization among Medical Aid recipients stemmed from their increased risk in stages 3 (aOR, 2.88; 95% CI, 2.85 to 2.92) and 4 (aOR, 2.52; 95% CI, 2.48 to 2.56), a trend mirrored by those with disabilities. Among that latter group, the risk of hospitalization was highest in stage 3 (aOR, 2.08; 95% CI, 2.06 to 2.10) and stage 4 (aOR, 2.04; 95% CI, 2.02 to 2.06) (Table 4).

Regarding factors associated with mortality, the risk of death was found to be lower in female (aOR, 0.58; 95% CI, 0.57 to 0.59) and in children and adolescents under 20 years old (aOR, 0.05; 95% CI, 0.04 to 0.06). In turn, the risk of death was sharply elevated in older adults, with an aOR of 9.82 (95% CI, 9.11 to 10.58) for those aged 65-75 years and an aOR of 215.05 for individuals aged 75 years and older (95% CI, 200.01 to 231.22). The fifth (lowest) level of economic status faced the highest risk of death (aOR relative to the first [highest] level, 1.92; 95% CI, 1.87 to 1.98), while also displaying the greatest aOR values from stage 1 to stage 4. Residing in a non-metropolitan area was similarly associated with an elevated risk of mortality (aOR, 1.06; 95% CI, 1.04 to 1.08), as were higher CCI scores (aOR, 2.25; 95% CI, 2.19 to 2.32 for CCI≥ 3) and having a disability (aOR, 1.65; 95% CI, 1.62 to 1.69). Vaccination was associated with a reduced risk of death, with an aOR of 0.09 (95% CI, 0.09 to 0.10) (Table 5).

## DISCUSSION

As the COVID-19 pandemic approaches its conclusion, a final report is necessary to encapsulate the impact of COVID-19 and inform responses to future public health crises. Our study utilized nationally representative data to examine the incidence rate, disease severity, mortality rate, and case-fatality rate throughout the pandemic, as well as to identify any disparities present based on socioeconomic status. The analysis was stratified by the stages of the COVID-19 pandemic period.

The results confirmed that as the pandemic progressed from the initial stages (1 and 2) to the latter phases (3 and 4), incidence, hospitalization, and mortality rates surged, and the disparities associated with economic status and disability became more pronounced. Conversely, the case-fatality rate was high during stage 1 but gradually decreased thereafter, indicating a weakening impact of the virus. Risk factors for COVID-19 hospitalization and death included male sex, advanced age, lower economic status, non-metropolitan area of residence, multiple comorbidities, disability, and incomplete vaccination.

This study confirmed that the incidence, hospitalization, and mortality rates increased sharply during stages 3 and 4. The rise in incidence can be attributed to changes in national policies, such as the relaxation of social distancing measures, and the emergence of the highly transmissible Omicron variant. As social distancing restrictions were lifted by the government, interpersonal contact increased and transmission-reducing behaviors declined, resulting in a high basic reproductive number (R0) [16,17]. Concurrently, the spread of the Omicron variant led to a surge in infections. Due to its greater infectivity and vaccine breakthrough capacity, the Omicron variant supplanted the Delta variant as the predominant strain of COVID-19, and the total number of infections with the Omicron variant rose [8].

Increasing COVID-19 case numbers led to a shortage of medical resources, contributing to rising mortality from the disease. As the pandemic progressed to stages 3 and 4, a marked rise was seen in the number of confirmed cases. This led to a surge in the number of hospitalized patients and a dearth of hospital beds and healthcare personnel. Despite the hospitalization of vulnerable populations, such as individuals with low economic status and those with disabilities, efforts to curb the rising mortality rates were hampered. Previous research has shown that vulnerable groups encounter more difficulties in adhering to social distancing guidelines, especially when living in densely populated areas [18]. This places them at a greater risk of infection, including with COVID-19. Additionally, the prevalence of pre-existing health conditions in these populations further exacerbates the severity of the disease [6,19].

Another factor contributing to increased mortality from COVID-19 is the withdrawal of UHC. At the beginning of the pandemic, Korea strengthened access to healthcare through UHC, eliminating out-of-pocket expenses for COVID-19 diagnosis and treatment nationwide [6]. However, as the pandemic progressed and patient numbers rose, out-of-pocket costs for COVID-19 testing, treatment, and medication also increased. This may have impacted the ability of vulnerable individuals to access healthcare, potentially widening the mortality gap. A study examining COVID-19 mortality in relation to out-of-pocket expenses across 179 countries found that countries with greater out-of-pocket costs experienced higher mortality rates [20]. Furthermore, disparities in access to healthcare facilities and the resulting outcomes have been reported to vary based on the type of insurance coverage, with these differences impacting COVID-19 mortality rates [21]. In the present study, individuals in the fifth (lowest) level of economic status were covered by Medical Aid insurance, which may have influenced disparities in healthcare access.

Despite the observed disparities, Korea maintained a low case-fatality rate across all economic groups. Korea’s overall incidence rate was not lower than the G7 national average of all populations during the same period (40,601 per 100,000 vs. 36,203 per 100,000, respectively); however, its mortality rate was less than one-sixth of that of the G7 countries (105 per 100,000 vs. 687 per 100,000) [22]. Even comparing the lowest economic level of Korea’s population with overall rates from the G7 countries, the group within Korea displayed a lower incidence (32,737 per 100,000 vs. 36,203 per 100,000) and lower mortality (498 per 100,000 vs. 687 per 100,000) [22]. During the early stages of the pandemic, Korea effectively controlled the viral spread on a national scale, thereby preventing large-scale outbreaks. In the latter era of the pandemic, when medical resources were scarce, Korea prioritized high-risk groups such as those with underlying diseases and older adults [7]. Furthermore, higher vaccination rates in Korea likely contributed to the relatively low mortality rates [23].

In the present study, the risk factors for hospitalization and death from COVID-19 aligned with those identified in previous research. Multiple studies have indicated that individuals with low socioeconomic status and those from ethnic minority groups are particularly vulnerable to severe COVID-19 outcomes [24]. Additionally, factors such as male sex [25,26], advanced age [25,27], the presence of comorbidities [25-27], and incomplete vaccination [28] have been associated with an increased risk of severe COVID-19 illness and mortality. These risk factors may shift depending on factors such as quarantine policies, vaccination rates, and the prevailing COVID-19 variant. Our study is meaningful in that it encompasses the entire 3-year span of the pandemic, providing comprehensive results.

This study has several limitations. First, it relies on insurance claims data, which means that patients with COVID-19 who did not seek medical care or who had asymptomatic infections were not captured [29,30]. While Korea conducted widespread screening of potential cases in the early stages of the pandemic, testing later became voluntary [31]. As a result, identifying individuals who did not seek medical care during the later stages was challenging. Second, the specific causes of death were not ascertainable in our research. Consequently, any death that occurred during hospitalization with COVID-19 listed as the primary diagnosis was recorded as a COVID-19 death. However, this approach raises the possibility that some deaths not caused by COVID-19 were included. Lastly, our study did not capture the clinical symptoms experienced by patients. This means that variations may exist in the clinical presentations among patients categorized at the same severity level.

In conclusion, Korea experienced a high incidence of COVID19 but a low mortality rate. Initially, the incidence of COVID-19 was low, with relatively high case-fatality. Nevertheless, no marked disparity in health outcomes was observed. As the pandemic evolved, its incidence, hospitalization, and mortality rates increased, which led to growing inequalities based on economic status and disability. These trends are likely attributable to changes in healthcare accessibility policies, including constraints on healthcare resources and rising out-of-pocket expenses. To address these issues, it is crucial to ensure sustained healthcare accessibility through UHC and to bolster the healthcare system’s preparedness for effectively managing future health crises.

## Figures and Tables

**Figure f1-epih-46-e2024038:**
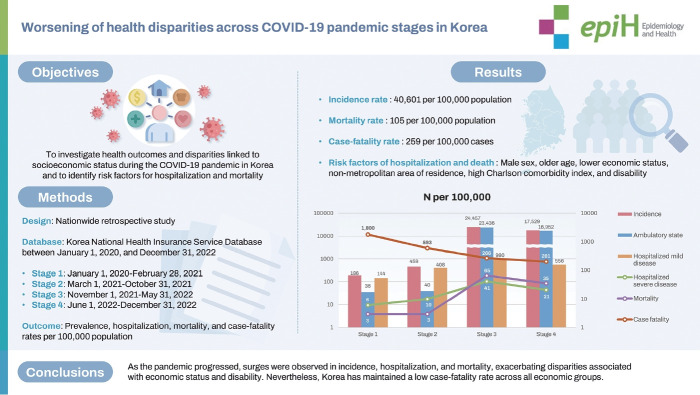


**Table 1.** General characteristics of Korean population according to COVID-19 infection status

Characteristics	Total population	Patients with confirmed COVID-19	Individuals who were not infected or confirmed to have COVID-19	p-value
Total	51,984,158 (100)	21,105,865 (100)	30,878,293 (100)	
Sex				<0.001
Male	26,006,437 (50.0)	9,493,140 (45.0)	16,513,297 (53.5)	
Female	25,977,721 (50.0)	11,612,725 (55.0)	14,364,996 (46.5)	
Age (yr)				<0.001
<20	8,584,915 (16.5)	4,733,369 (22.4)	3,851,546 (12.5)	
20-44	17,777,188 (34.2)	7,536,232 (35.7)	10,240,956 (33.2)	
45-64	17,058,292 (32.8)	5,896,422 (27.9)	11,161,870 (36.2)	
65-74	4,858,646 (9.4)	1,785,583 (8.5)	3,073,063 (10.0)	
≥75	3,705,117 (7.1)	1,154,259 (5.5)	2,550,858 (8.3)	
Level of economic status				<0.001
First (highest)	17,343,806 (33.4)	7,571,375 (35.9)	9,772,431 (31.7)	
Second	12,954,991 (24.9)	5,336,097 (25.3)	7,618,894 (24.7)	
Third	10,329,060 (19.9)	4,021,754 (19.1)	6,307,306 (20.4)	
Fourth	9,868,764 (19.0)	3,689,668 (17.5)	6,179,096 (20.0)	
Medical Aid (lowest)	1,487,537 (2.9)	486,971 (2.3)	1,000,566 (3.2)	
Region				<0.001
Metropolitan area	36,399,732 (70.0)	15,022,546 (71.2)	21,377,186 (69.2)	
Non-metropolitan area	15,584,426 (30.0)	6,083,319 (28.8)	9,501,107 (30.8)	
Charlson comorbidity index				<0.001
0	28,715,680 (55.2)	11,265,937 (53.4)	17,449,743 (56.5)	
1-2	17,551,382 (33.8)	7,703,605 (36.5)	9,847,777 (31.9)	
≥3	5,717,096 (11.0)	2,136,323 (10.1)	3,580,773 (11.6)	
Disability				<0.001
No	49,292,720 (94.8)	20,189,078 (95.7)	29,103,642 (94.3)	
Yes	2,691,438 (5.2)	916,787 (4.3)	1,774,651 (5.8)	
Vaccination				<0.001
Incomplete	9,130,293 (17.6)	3,891,913 (18.4)	5,238,380 (17.0)	
Complete	42,853,865 (82.4)	17,213,952 (81.6)	25,639,913 (83.0)	
Comorbidities				
Hypertension	9,568,805 (18.4)	3,361,222 (15.9)	6,207,583 (20.1)	<0.001
Diabetes mellitus	5,035,927 (9.7)	1,806,420 (8.6)	3,229,507 (10.5)	<0.001
Dyslipidemia	11,604,115 (22.3)	4,453,247 (21.1)	7,150,868 (23.2)	<0.001
Myocardial infarction	199,029 (0.4)	67,698 (0.3)	131,331 (0.4)	<0.001
Stroke	979,728 (1.9)	334,650 (1.6)	645,078 (2.1)	<0.001
Cancer	1,898,331 (3.7)	742,971 (3.5)	1,155,360 (3.7)	<0.001
Severity of infection				<0.001
Ambulatory state	20,011,285 (38.5)	20,011,285 (94.8)	-	
Hospitalized mild disease	1,054,809 (2.0)	1,054,809 (5.0)	-	
Hospitalized severe disease	39,771 (0.1)	39,771 (0.2)	-	
Instances of COVID-19 infection				<0.001
0	30,878,293 (59.4)	-	30,878,293 (100)	
1	20,325,112 (39.1)	20,325,112 (96.3)	-	
2	776,760 (1.5)	776,760 (3.7)	-	
≥3	3,993 (0.0)	3,993 (0.0)	-	

Values are presented as number (%); Percentages do not necessarily total 100 because of rounding. COVID-19, coronavirus disease 2019.

**Table 2.** Cumulative incidence, severity, and case-fatality of COVID-19 by stage^1^

Variables	Total		Stage 1		Stage 2		Stage 3		Stage 4	
	n^2^	No. per 100,000	n^2^	No. per 100,000	n^2^	No. per 100,000	n^2^	No. per 100,000	n^2^	No. per 100,000
Incidence	21,105,865	40,601	96,889	186	236,870	459	12,577,367	24,457	8,979,635	17,529
Severity of infection										
Ambulatory state	20,011,285	38,495	18,795	36	20,807	40	12,052,216	23,436	8,684,297	16,952
Hospitalized mild disease	1,054,809	2,029	75,041	144	210,886	408	504,104	980	284,608	556
Hospitalized severe disease	39,771	77	3,053	6	5,177	10	21,047	41	10,730	21
Mortality	54,638	105	1,744	3	1,405	3	33,404	65	18,085	35
Case-fatality	54,638	259	1,744	1,800	1,405	593	33,404	266	18,085	201

COVID-19, coronavirus disease 2019.

1 Stage 1 covers January 1, 2020 to February 28, 2021; Stage 2 covers March 1, 2021 to October 31, 2021; Stage 3 covers November 1, 2021 to May 31, 2022; and Stage 4 covers June 1, 2022 to December 31, 2022; Stage 1 represents the period from the first confirmed case to the issuance of a serious-level infection disease crisis alert; Stage 2 corresponds to the time when social distancing and quarantine measures were strengthened and vaccination efforts began; Stage 3 marks the start of a gradual daily recovery; while Stage 4 signifies an attempt to achieve full daily recovery.

2 Signifies a cumulative number for each stage, spanning a total duration of 3 years; The overall period covers 3 years, with stage 1 spanning 14 months, stage 2 covering 8 months, and stages 3 and 4 lasting 7 months each.

**Table 3.** Cumulative incidence, severity, and case-fatality of COVID-19 according to stage, economic status, and disability^1^

Variables	Total			Stage 1			Stage 2			Stage 3			Stage 4		
	n^2^	No. per 100,000	p-value	n^2^	No. per 100,000	p-value	n^2^	No. per 100,000	p-value	n^2^	No. per 100,000	p-value	n^2^	No. per 100,000	p-value
Incidence															
Level of economic status			<0.001			<0.001			<0.001			<0.001			<0.001
First (highest)	7,571,375	43,655		29,987	173		71,442	415		4,466,956	26,030		3,285,025	19,217	
Second	5,336,097	41,190		23,031	178		60,602	470		3,215,248	25,021		2,237,648	17,463	
Third	4,021,754	38,936		19,946	193		52,146	508		2,385,813	23,294		1,708,733	16,733	
Fourth	3,689,668	37,387		19,050	193		46,332	473		2,211,328	22,651		1,547,483	15,913	
Medical Aid (lowest)	486,971	32,737		4,875	328		6,348	441		298,022	21,126		200,746	14,508	
Disability			<0.001			<0.001			<0.001			<0.001			<0.001
No	20,189,078	40,958		89,482	182		227,463	464		12,027,375	24,604		8,594,790	17,635	
Yes	916,787	34,063		7,407	275		9,407	362		549,992	21,622		384,845	15,458	
Hospitalization															
Level of economic status			<0.001			<0.001			<0.001			<0.001			<0.001
First (highest)	357,131	2,059		24,600	142		65,798	382		171,318	998		101,613	594	
Second	250,656	1,935		18,201	140		54,995	427		115,632	900		65,611	512	
Third	200,327	1,939		15,854	153		47,287	460		90,649	885		49,433	484	
Fourth	202,221	2,049		15,413	156		42,365	432		96,374	987		51,812	533	
Medical Aid (lowest)	84,245	5,663		4,026	271		5,618	391		51,178	3,628		26,869	1,942	
Disability			<0.001			<0.001			<0.001			<0.001			<0.001
No	942,803	1,913		72,137	146		207,687	424		435,089	890		241,507	496	
Yes	151,777	5,639		5,957	221		8,376	322		90,062	3,541		53,831	2,162	
Hospitalized with severe disease															
Level of economic status			<0.001			<0.001			<0.001			<0.001			<0.001
First (highest)	14,139	82		1,125	6		1,758	10		7,307	43		4,031	24	
Second	7,989	62		591	5		1,144	9		4,189	33		2,125	17	
Third	6,322	61		480	5		1,010	10		3,299	32		1,565	15	
Fourth	6,893	70		543	6		927	9		3,705	38		1,759	18	
Medical Aid (lowest)	4,428	298		314	21		338	24		2,547	181		1,250	90	
Disability			<0.001			<0.001			<0.001			<0.001			<0.001
No	28,718	58		2,398	5		4,465	9		14,762	30		7,268	15	
Yes	11,053	411		655	24		712	27		6,285	247		3,462	139	
Mortality															
Level of economic status			<0.001			<0.001			<0.001			<0.001			<0.001
First (highest)	19,206	111		606	3		494	3		11,514	67		6,592	39	
Second	10,095	78		310	2		258	2		6,117	48		3,410	27	
Third	7,865	76		258	2		220	2		4,803	47		2,584	25	
Fourth	10,065	102		308	3		250	3		6,244	64		3,263	34	
Medical Aid (lowest)	7,407	498		262	18		183	13		4,726	335		2,236	162	
Disability			<0.001			<0.001			<0.001			<0.001			<0.001
No	38,360	78		1,263	3		1,058	2		23,209	47		12,830	26	
Yes	16,278	605		481	18		347	13		10,195	401		5,255	211	
Case-fatality															
Level of economic status			<0.001			<0.001			<0.001			<0.001			<0.001
First (highest)	19,206	254		606	2,021		494	691		11,514	258		6,592	201	
Second	10,095	189		310	1,346		258	426		6,117	190		3,410	152	
Third	7,865	196		258	1,293		220	422		4,803	201		2,584	151	
Fourth	10,065	273		308	1,617		250	540		6,244	282		3,263	211	
Medical Aid (lowest)	7,407	1521		262	5,374		183	2,883		4,726	1,586		2,236	1,114	
Disability			<0.001			<0.001			<0.001			<0.001			<0.001
No	38,360	190		1,263	1,411		1,058	465		23,209	193		12,830	149	
Yes	16,278	1,776		481	6,494		347	3,689		10,195	1,854		5,255	1,365	

COVID-19, coronavirus disease 2019.

1 Stage 1 covers January 1, 2020 to February 28, 2021; Stage 2 covers March 1, 2021 to October 31, 2021; Stage 3 covers November 1, 2021 to May 31, 2022; and Stage 4 covers June 1, 2022 to December 31, 2022; Stage 1 represents the period from the first confirmed case to the issuance of a serious-level infection disease crisis alert; Stage 2 corresponds to the time when social distancing and quarantine measures were strengthened and vaccination efforts began; Stage 3 marks the start of a gradual daily recovery; while Stage 4 signifies an attempt to achieve full daily recovery.

2 Signifies a cumulative number for each stage, spanning a total duration of 3 years; The overall period covers 3 years, with stage 1 spanning 14 months, stage 2 covering 8 months, and stages 3 and 4 lasting 7 months each.

**Table 4.** Factors associated with COVID-19 hospitalization^1^

Variables	Total	Stage 1	Stage 2	Stage 3	Stage 4
Sex					
Male	1.00 (reference)	1.00 (reference)	1.00 (reference)	1.00 (reference)	1.00 (reference)
Female	0.84 (0.84, 0.84)	0.95 (0.92, 0.99)	0.92 (0.89, 0.94)	0.82 (0.82, 0.83)	0.98 (0.97, 0.99)
Age (yr)					
<20	0.64 (0.64, 0.65)	0.82 (0.78, 0.87)	0.83 (0.79, 0.86)	0.66 (0.65, 0.67)	0.89 (0.88, 0.90)
20-44	1.00 (reference)	1.00 (reference)	1.00 (reference)	1.00 (reference)	1.00 (reference)
45-64	1.32 (1.31, 1.33)	2.06 (1.97, 2.14)	1.21 (1.17, 1.26)	1.37 (1.36, 1.38)	1.73 (1.70, 1.75)
65-74	1.99 (1.98, 2.00)	2.35 (2.21, 2.50)	0.97 (0.91, 1.03)	2.41 (2.38, 2.43)	3.30 (3.25, 3.34)
≥75	5.17 (5.13, 5.21)	2.06 (1.92, 2.21)	0.99 (0.91, 1.07)	6.91 (6.84, 6.98)	9.32 (9.19, 9.45)
Level of economic status					
First (highest)	1.00 (reference)	1.00 (reference)	1.00 (reference)	1.00 (reference)	1.00 (reference)
Second	1.12 (1.11, 1.13)	0.86 (0.82, 0.89)	0.83 (0.80, 0.86)	1.08 (1.07, 1.09)	1.16 (1.15, 1.17)
Third	1.23 (1.22, 1.24)	0.87 (0.83, 0.91)	0.81 (0.78, 0.84)	1.18 (1.17, 1.19)	1.22 (1.20, 1.23)
Fourth	1.27 (1.26, 1.28)	0.93 (0.89, 0.97)	0.88 (0.85, 0.92)	1.25 (1.24, 1.26)	1.24 (1.23, 1.25)
Medical Aid (lowest)	2.55 (2.53, 2.58)	1.05 (0.97, 1.15)	0.72 (0.66, 0.78)	2.88 (2.85, 2.92)	2.52 (2.48, 2.56)
Region					
Metropolitan area	1.00 (reference)	1.00 (reference)	1.00 (reference)	1.00 (reference)	1.00 (reference)
Non-metropolitan area	1.07 (1.06, 1.07)	1.17 (1.12, 1.23)	2.49 (2.37, 2.62)	1.10 (1.09, 1.10)	1.80 (1.78, 1.81)
Charlson comorbidity index					
0	1.00 (reference)	1.00 (reference)	1.00 (reference)	1.00 (reference)	1.00 (reference)
1-2	1.07 (1.06, 1.07)	0.78 (0.75, 0.81)	0.88 (0.85, 0.91)	1.08 (1.07, 1.09)	1.33 (1.32, 1.34)
≥3	1.59 (1.58, 1.60)	0.50 (0.48, 0.53)	0.75 (0.71, 0.80)	1.69 (1.67, 1.70)	2.20 (2.17, 2.22)
Disability					
No	1.00 (reference)	1.00 (reference)	1.00 (reference)	1.00 (reference)	1.00 (reference)
Yes	1.85 (1.83, 1.86)	0.86 (0.81, 0.92)	0.80 (0.75, 0.86)	2.08 (2.06, 2.10)	2.04 (2.02, 2.06)
Vaccination					
Incomplete	1.00 (reference)	-	1.00 (reference)	1.00 (reference)	1.00 (reference)
Complete	0.40 (0.39, 0.40)	-	1.14 (1.10, 1.18)	0.37 (0.36, 0.37)	0.31 (0.31, 0.32)

Values are presented as adjusted odds ratio (95% confidence interval); Multivariable logistic regression included sex, age, economic status, region, Charlson comorbidity index, disability, and vaccination status; Vaccination was not performed during stage 1, so odds ratios for this variable could not be calculated. COVID-19, coronavirus disease 2019.

1 Stage 1 covers January 1, 2020 to February 28, 2021; Stage 2 covers March 1, 2021 to October 31, 2021; Stage 3 covers November 1, 2021 to May 31, 2022; and Stage 4 covers June 1, 2022 to December 31, 2022; Stage 1 represents the period from the first confirmed case to the issuance of a serious-level infection disease crisis alert; Stage 2 corresponds to the time when social distancing and quarantine measures were strengthened and vaccination efforts began; Stage 3 marks the start of a gradual daily recovery; while Stage 4 signifies an attempt to achieve full daily recovery.

**Table 5.** Factors associated with COVID-19 case-fatality^1^

Variables	Total	Stage 1	Stage 2	Stage 3	Stage 4
Sex					
Male	1.00 (reference)	1.00 (reference)	1.00 (reference)	1.00 (reference)	1.00 (reference)
Female	0.58 (0.57, 0.59)	0.63 (0.56, 0.71)	0.62 (0.55, 0.70)	0.60 (0.58, 0.61)	0.55 (0.53, 0.56)
Age (yr)					
<20	0.05 (0.04, 0.06)	0.04 (0.00, 0.30)	<0.01 (<0.01->99.99)	0.04 (0.03, 0.05)	0.08 (0.06, 0.11)
20-44	1.00 (reference)	1.00 (reference)	1.00 (reference)	1.00 (reference)	1.00 (reference)
45-64	9.82 (9.11, 10.58)	9.14 (5.44, 15.35)	11.32 (8.06, 15.91)	9.98 (9.04, 11.02)	9.29 (8.18, 10.54)
65-74	40.83 (37.91, 43.97)	36.22 (21.65, 60.57)	46.26 (32.73, 65.39)	42.42 (38.47, 46.75)	37.42 (33.02, 42.39)
≥75	215.05 (200.01, 231.22)	128.25 (77.27, 212.86)	177.33 (126.28, 249.00)	223.83 (203.46, 246.24)	204.78 (181.28, 231.32)
Level of economic status					
First (highest)	1.00 (reference)	1.00 (reference)	1.00 (reference)	1.00 (reference)	1.00 (reference)
Second	1.16 (1.13, 1.18)	0.99 (0.83, 1.17)	0.93 (0.79, 1.10)	1.13 (1.09, 1.16)	1.21 (1.16, 1.26)
Third	1.23 (1.20, 1.26)	1.03 (0.85, 1.23)	1.03 (0.86, 1.22)	1.20 (1.16, 1.24)	1.28 (1.22, 1.33)
Fourth	1.36 (1.32, 1.39)	1.09 (0.92, 1.30)	1.03 (0.87, 1.22)	1.31 (1.27, 1.35)	1.41 (1.35, 1.47)
Medical Aid (lowest)	1.92 (1.87, 1.98)	1.22 (1.01, 1.48)	1.34 (1.09, 1.65)	1.87 (1.81, 1.94)	2.00 (1.90, 2.11)
Region					
Metropolitan area	1.00 (reference)	1.00 (reference)	1.00 (reference)	1.00 (reference)	1.00 (reference)
Non-metropolitan area	1.06 (1.04, 1.08)	0.94 (0.81, 1.08)	0.81 (0.69, 0.95)	1.05 (1.02, 1.08)	1.18 (1.14, 1.21)
Charlson comorbidity index					
0	1.00 (reference)	1.00 (reference)	1.00 (reference)	1.00 (reference)	1.00 (reference)
1-2	1.48 (1.43, 1.52)	2.07 (1.70, 2.52)	1.53 (1.29, 1.81)	1.47 (1.41, 1.52)	1.41 (1.33, 1.48)
≥3	2.25 (2.19, 2.32)	2.28 (1.88, 2.78)	2.45 (2.06, 2.91)	2.22 (2.14, 2.30)	2.16 (2.05, 2.26)
Disability					
No	1.00 (reference)	1.00 (reference)	1.00 (reference)	1.00 (reference)	1.00 (reference)
Yes	1.65 (1.62, 1.69)	1.24 (1.07, 1.43)	1.41 (1.21, 1.64)	1.64 (1.60, 1.69)	1.67 (1.61, 1.73)
Vaccination					
Incomplete	1.00 (reference)	-	1.00 (reference)	1.00 (reference)	1.00 (reference)
Complete	0.09 (0.09, 0.10)	-	0.03 (0.03, 0.04)	0.10 (0.09, 0.10)	0.14 (0.14, 0.15)

Values are presented as adjusted odds ratio (95% confidence interval); Multivariable logistic regression included sex, age, level of economic status, region, Charlson comorbidity index, disability, and vaccination status; Vaccination was not performed during stage 1, so odds ratios for this variable could not be calculated. COVID-19, coronavirus disease 2019.

1 Stage 1 covers January 1, 2020 to February 28, 2021; Stage 2 covers March 1, 2021 to October 31, 2021; Stage 3 covers November 1, 2021 to May 31, 2022; and Stage 4 covers June 1, 2022 to December 31, 2022; Stage 1 represents the period from the first confirmed case to the issuance of a serious-level infection disease crisis alert; Stage 2 corresponds to the time when social distancing and quarantine measures were strengthened and vaccination efforts began; Stage 3 marks the start of a gradual daily recovery; while Stage 4 signifies an attempt to achieve full daily recovery.
